# Who is serving whom? Exploring the mechanisms linking technology dependence to work engagement

**DOI:** 10.3389/fpsyg.2025.1494173

**Published:** 2025-02-19

**Authors:** Arūnas Žiedelis, Ieva Urbanavičiūtė, Jurgita Lazauskaitė-Zabielskė, Rita Jakštienė

**Affiliations:** Organizational Psychology Research Center, Vilnius University, Vilnius, Lithuania

**Keywords:** basic need satisfaction, autonomy, learning demands, technology dependence, work engagement

## Abstract

As technological advances increasingly shape the world of work, it is becoming clear that the impact of increased employee dependence on technology for task completion may have ambivalent effects. While successfully mastered technological tools make the work more engaging by enabling a simpler and more efficient implementation of tasks, the necessity to keep up with technological progress imposes additional demands to adopt innovations and limits the freedom of choice about how work is performed. With this study, we sought to unravel whether the effect of technology dependence at work on employee autonomy satisfaction and subsequent work engagement is conditional and depends on increased learning demands. A heterogeneous sample of 753 employees participated in the survey. We used conditional process analysis to test our hypotheses. Results revealed that it is not dependence on technology itself that is relevant for autonomy and work engagement, but rather its combination with intensified learning demands. For employees who reported fewer learning pressures, technology dependence was an enabling (i.e., facilitating autonomy satisfaction) factor, associated with higher work engagement. Conversely, for those who experienced greater learning demands, technology dependence was associated with lower autonomy satisfaction and lower work engagement.

## 1 Introduction

Innovative technologies are becoming an increasingly integral part of work. The COVID-19 pandemic has accelerated the shift to telecommuting, 3D printers are opening up new opportunities in fields ranging from construction to healthcare, 5G technologies are enabling faster development of the Internet of Things, and machine learning algorithms are already capable of performing various types of creative activities (Eurofound, 2021). However, technological progress, including the rise of artificial intelligence and robotics, might not only relieve employees from the need to perform routine activities but also pose a threat to their position in the labor market (Oosthuizen, 2022). A recent review by the World Economic Forum (2023) revealed that the latest technologies are responsible for both the demand for personnel in the fastest-growing professions and the lack of demand for employees in the fastest-shrinking occupations. Thus, emerging technological developments in the workplace have the potential to both empower workers to be more productive and out-compete them in the unequal battle for productivity (Eurofound, 2021).

These trends have sparked growing research interest in better understanding the technological dimension of work and its psychological effects. As a result, various phenomena associated with the use of technology, such as increased workload (Graf and Antoni, 2023), social disconnection (Collins et al., 2016), and technostress (Bondanini et al., 2020), have been extensively studied. However, to date, researchers have paid limited attention to the phenomenon of technology dependence (i.e., the necessity to use technology to perform work tasks; Karr-Wisniewski and Lu, 2010), and to our knowledge, there has been no empirical research on the relationship between technology dependence and employee autonomy. This gap in the literature needs to be addressed, as the growing reliance on technology is a key driver of transformation in modern organizations (Verhoef et al., 2021). With the acceleration of technological advancements, new technologies are becoming an inevitable part of work across many professional fields, bringing both positive and negative consequences (Gagné et al., 2022; Parker and Grote, 2022). New technologies can simplify or even eliminate monotonous tasks and facilitate flexible work arrangements, which positively impacts employee autonomy and work engagement. On the other hand, they can also increase the need to focus on the tools themselves, thereby increasing job demands, or limit work to the passive supervision of ongoing processes, reducing opportunities for employees to utilize their skills and make autonomous decisions (Gagné et al., 2022; Parker and Grote, 2022). Given the dual impact of technological progress, it is crucial to understand the conditions under which dependence on technology enhances and motivates employees and when such reliance frustrates their basic needs, leading to decreased work engagement.

In this study, we explore a fundamental question about the relationship between technology dependence, autonomy satisfaction, and work engagement by investigating the determinants of agency within the employee-technology dyad. In other words, our aim is to clarify what determines whether employees perceive technology as an enabling factor that serves them and supports their basic need for autonomy or as a force that makes them feel like servants to technology themselves. In doing so, our results offer several contributions to the existing literature. First, we show that dependence on technology can be related to both the satisfaction and frustration of the basic need for autonomy, depending on the extent to which employees feel exposed to intensified learning demands. In other words, our study highlights that the frustration of autonomy is not due to dependence on technology per se but rather to its interaction with intensifying learning demands. In this way, we respond to repeated calls in the literature (Parker and Grote, 2022; see also Gagné et al., 2022) to better understand the impact of new technologies on employee autonomy and work-based need satisfaction. Second, we show that the combined effect of technology dependence and learning demands on autonomy remains relevant in predicting work engagement. As such, the question of who is serving whom has a conceptual and practical meaning related to employees’ daily functioning and performance. Third, the results of our study reveal that the need for autonomy is most likely to be satisfied among employees who experience high learning demands but have low dependence on technology at work. This further illustrates that the impact of technological progress on employee outcomes is shaped by broader contextual factors.

### 1.1 Technology dependence and employee autonomy

Self-Determination Theory (SDT; Ryan and Deci, 2002) proposes that people are internally predisposed toward greater integration, growth, and well-being. However, to achieve this, they need to satisfy their basic psychological needs for autonomy, competence, and relatedness. The need for autonomy is satisfied when individuals can act in alignment with their authentic interests and values. The need for competence is satisfied through feelings of mastery and effectiveness, whereas relatedness refers to the universal need for social connection, belonging, feeling cared for, and contributing to others. The satisfaction of basic needs during the performance of an activity promotes vitality and encourages the internalization of its motives (Autin et al., 2022; Ryan and Deci, 2017). Therefore, feeling autonomous, competent, and connected to others at work fosters work engagement (Schaufeli, 2021).

Among the three basic needs, autonomy has been the most extensively researched (Ryan and Deci, 2017). Usually, the levels of support and satisfaction for autonomy and the other two basic psychological needs are strongly correlated, with at least two known explanations for this. First, organizational and work environments that support autonomy tend to foster feelings of competence and relatedness among employees. When managers and organizational cultures prioritize autonomy, employees are more likely to feel capable and connected to their colleagues (Deci et al., 2017). Second, when employees feel that their autonomy is supported, they are better able to satisfy their other basic needs. For example, they can choose tasks and perform them in ways that enhance their sense of mastery and maintain positive relationships with coworkers (Deci et al., 2017). Therefore, the satisfaction of autonomy serves as a good indicator of the overall satisfaction of basic psychological needs, which, in turn, predicts greater work engagement.

By definition, work engagement is a positive work-related state characterized by vigor, dedication, and absorption (Schaufeli et al., 2002). Engaged employees have high energy levels, are resilient, willing to invest effort, and persist when difficulties arise. Moreover, they perceive their work as significant, inspiring, and challenging. Finally, they are concentrated and engrossed in their work to the extent that they frequently find it difficult to detach from work activities (Schaufeli and Bakker, 2010). Thus, employees who feel autonomous are more energetic and dedicated to their work, whereas the frustration of autonomy is associated with lower work engagement (Shuck et al., 2015).

Technology dependence refers to the extent to which technology is indispensable for the successful completion of work tasks (Karr-Wisniewski and Lu, 2010). Information and communication technologies become more and more sophisticated and the increasing reliance on them for work tasks is one of the main factors shaping the dynamics of the modern world of work (Gagné et al., 2022; Verhoef et al., 2021). As more tasks become inseparable from the use of technology, this dependence – like all forms of dependence – may seem incompatible with employees’ need for autonomy and, therefore, could be seen as a demotivating factor reducing work engagement. However, as Ryan and Lynch (1989) aptly observed, autonomy and independence are separate and not necessarily related phenomena. Autonomy refers to self-regulation, and this need can be satisfied by enacting behavior that is either self-initiated or endorsed by oneself (Ryan and Deci, 2017). On the other hand, independence refers to self-reliance, being able to take care of oneself and achieve one’s goals without the help of others (Ryan and Lynch, 1989). Therefore, employees can feel autonomous while being dependent on someone (e.g., cooperating in activities they fully support) and vice versa (e.g., independently performing activities that conflict with their goals and values).

Digit(al)ization and the digital transformation of organizations mean, among other things, that employees are becoming increasingly dependent not only on other individuals (which is universal to humans from an evolutionary point of view) but also on digital tools and systems (Verhoef et al., 2021). The notion that such dependence on technology at work (and technology use in general) is a double-edged sword for one’s functioning in the workplace is well-established in the literature (Gagné et al., 2022; Marsh et al., 2022), with several studies supporting the idea that it can both satisfy and frustrate autonomy. Bloom et al.'s (2014) research showed that technologies that facilitate the dissemination of information promote more decentralized decision-making within an organization, whereas communication technologies promote decision-making at higher levels of management. In this way, the set of technologies implemented in the organization influences the level of decision latitude available to employees. A study by Lehdonvirta (2018) revealed that online piecework platform workers have greater control over their schedules, but some find it challenging to use due to a lack of self-control. As a result, these employees experience procrastination and unproductive presenteeism – issues far less common in traditionally organized work – that undermine their autonomy. Finally, numerous studies examining the impact of technology-enabled remote work on the well-being of employees also point out that such work arrangements can both increase flexibility in terms of working time and place and impose constraints due to the emerging need for constant availability or the presence of algorithmic management (Gagné et al., 2022; Wang et al., 2020). Thus, technological development does not necessarily translate into improved (or reduced) employee well-being by itself, which calls for a better understanding of the factors moderating this relationship.

### 1.2 Intensified learning demands

The primary argument for integrating new technologies into work processes is that they increase efficiency and facilitate the achievement of organizational goals. However, technological progress is also associated with the shortening half-life of information, forcing individuals to constantly update their knowledge and skills to remain competitive in the labor market (Eurofound, 2021). Kubicek et al. (2015) proposed the concept of intensified learning demands as a side effect of technological progress that impacts employees. This concept highlights the idea that, due to continuous advancements in technology, employees are forced to constantly update their knowledge and skills to remain effective and competitive at work. Otherwise, they risk becoming redundant in the ever-changing labor market (Dellot and Wallace-Stephens, 2017). For this reason, learning demands refer to the necessity rather than a self-initiated and non-binding incentive to develop one’s skills. They are conceptually distinct from the newly suggested concept of “new learning” (Decius et al., 2022), which denotes the intention of a proactive learner to take advantage of newly opened learning opportunities, thereby acquiring new competencies. In contrast, learning demands reflect external pressure to update qualifications due to technological progress, irrespective of the employee’s preferences for self-improvement (Kubicek et al., 2015). Thus, although both concepts may encompass similar learning behaviors, the employee’s role in the learning process (reactive vs. proactive) and the resulting implications for satisfying the need for autonomy are radically different.

Moreover, learning demands are not solely based on normative grounds, as the skill set an employee possesses differentiates those empowered by technology from those who are not, a phenomenon referred to as the “skills-based schism” (Parker and Grote, 2022). Previous research shows that technology affects tasks, schedule autonomy, and overall working conditions differently depending on an employee’s skill level. Higher-skilled employees, who perform less routine and more complex tasks that are harder to automate, can leverage information and communication technologies to eliminate monotonous activities, resulting in more satisfying and engaging work (Turja et al., 2024). In contrast, lower-skilled employees face a greater risk from technology-enabled automation, which often leads to the standardization of tasks and a shift from active skill utilization to passive oversight of automated processes (Parker and Grote, 2022). Additionally, research on remote work indicates that communication technologies enable flexible working practices, which, for lower-skilled workers, often translate into increased expectations for constant availability, chaotic schedules, and reduced autonomy over their work hours (Sewell and Taskin, 2015; Spreitzer et al., 2017). Finally, a meta-analysis by Parent-Rocheleau and Parker (2022) showed that the impact of algorithms on working conditions is moderated by employees’ ability to control the systems, provide input to them, or disengage from them—capabilities that are often lacking among those without the necessary skills. Hence, as technology becomes more prevalent in the work environment, the gap between employees with the right skills and those with high learning demands is widening.

### 1.3 Interrelation of technology dependence, autonomy satisfaction, and work engagement

In this study, we investigate the idea that intensified learning demands may determine the valence of the effects technology dependence has on autonomy and motivational outcomes (i.e., work engagement). Our reasoning is based on the following rationale. First, SDT posits that satisfying basic psychological needs is essential for individuals to internalize the motives for performing activities (Ryan and Deci, 2017). As a result, the impact of various environmental factors on employee motivation is mediated by the need for autonomy, competence, and relatedness. In other words, employees who feel autonomous, competent, and engaged in meaningful relationships are more likely to perform work for motives that align with their sense of self (Deci et al., 2017; Ryan and Deci, 2017). This internalization of motives promotes autonomous (rather than controlled) motivation and work engagement (Deci et al., 2017; Schaufeli, 2021). Among the basic needs, autonomy has been the most extensively researched, and it is thought that autonomy satisfaction enables employees to also meet their needs for competence and relatedness (Deci et al., 2017).

Second, as the world of work evolves and workers become increasingly dependent on various technologies (e.g., software enabling task performance), such changes can have a dual impact on autonomy. According to SDT, the relationship between autonomy and various (inter)dependencies (e.g., dependence on other people or things, such as technology) depends on the extent to which those dependencies are perceived as conflicting with personal goals, interests, and values (Ryan and Deci, 2017; Ryan and Lynch, 1989). This suggests that dependence on technology at work can satisfy the need for autonomy if the technologies are perceived as facilitating a more straightforward and efficient achievement of goals. Conversely, the same technology dependence can frustrate autonomy when it imposes externally created obligations, requiring employees to make additional efforts to keep up with the changing world of work. Empirical research also shows that new technologies can alter the work environment both positively and negatively in terms of employee autonomy (Bloom et al., 2014; Gagné et al., 2022; Lehdonvirta, 2018; Wang et al., 2020). Thus, increasing reliance on technology at work can either support and frustrate employees’ need for autonomy, depending on other factors.

Third, we hypothesize that one such factor is learning demands imposed by technology. In this regard, we agree with Parker and Grote (2022), who describe a “skill-based schism,” where technology empowers workers with the necessary skills while reducing the autonomy of those without these skills by negatively affecting their work content, schedule, and overall working conditions (Parent-Rocheleau and Parker, 2022; Sewell and Taskin, 2015; Spreitzer et al., 2017).

Building on these assumptions, we propose that intensified learning demands determine how dependence on technology will be perceived (as enabling or constraining) and thus act as a boundary condition in the relationship between this type of dependence on autonomy satisfaction and subsequent work engagement. Given that intensified learning demands are, by definition, driven by the external necessity to keep pace with ever-accelerating economic progress (Kubicek et al., 2015), their effect on employee behavior depends primarily on threat-avoidance (i.e., extrinsic) incentives, leading to externally regulated (and thus non-autonomous) behavior (Ryan and Deci, 2017). As a result, the combination of high reliance on technology and high learning demands is expected to frustrate the need for autonomy and relate to reduced work engagement. Conversely, reliance on technologies that are well-mastered and therefore do not require additional learning should help employees achieve their goals, thereby maintaining autonomy and increasing work engagement.

Based on this reasoning, we raise the following hypotheses.

*H1*: Technology dependence will have a different predictive effect on autonomy satisfaction depending on the level of intensified learning demands:

*H1a*: Among employees with low learning demands, technology dependence will positively predict autonomy satisfaction.

*H1b*: Among employees with high learning demands, technology dependence will negatively predict autonomy satisfaction.

*H2b*: Depending on the level of intensified learning demands, technology dependence will have a different predictive effect on work engagement through autonomy satisfaction:

*H2a*: Among employees with low learning demands, technology dependence will positively predict work engagement through autonomy satisfaction.

*H2b*: Among employees with high learning demands, technology dependence will negatively predict work engagement through autonomy satisfaction.

## 2 Research methods

### 2.1 Participants and procedure

The study was conducted from December 2022 to February 2023. The data was collected in Lithuania, a country experiencing rapid progress in many digitalization indicators. Almost half (49%) of its population possesses basic digital skills (compared to 54% in the EU), and nearly a quarter (23%) has above-basic digital skills (compared to 26% in the EU) (European Commission, 2024).

An online questionnaire was distributed to potential respondents with the help of student research assistants. All procedures performed in this study were in accordance with the 1964 Helsinki Declaration and its later amendments. Approval to conduct the study was also obtained from the institutional committee on research ethics in psychology. When invited to participate in the study, the potential participants were presented with the purpose of the study, informed about the use of data, and reminded of the right to refuse to take part in the survey at any time. They were also asked to indicate their agreement with the informed consent statement before proceeding to survey questions.

A heterogeneous convenience sample of 753 employees (210 males, 539 females, 4 indicated their gender identity as “other”) completed the questionnaire. The age of the study participants varied from 18 to 68 years (*M* = 30.7, SD = 9.0). The majority of the participants (82.5%) had obtained higher education, 83.4% worked full-time, and 90.3% worked under an open-ended employment contract. In terms of professional activity, 20.8% worked in the education and science sector, 12.1%—in the finance and insurance sector, 10.2% – in the information and communication sector, and 10.0%—in the trade sector. Moreover, regarding their working arrangements, 10.0% of the participants worked exclusively remotely, 43.0% worked only in the office, and the rest worked in a hybrid way. In terms of the hierarchical level, 20.2% of the subjects held managerial positions.

### 2.2 Measures

Respondents were asked to provide background information (gender, age, highest obtained education, work sector, type of contract, remote work, managerial position) and fill out an online questionnaire, among others, including items to assess technology dependence, intensified learning demands, basic need satisfaction at work, and work engagement. The internal consistency coefficients (Cronbach’s alpha) for all scales are presented in Table 1.

**Table 1:** Fit indices of alternative measurement models.

Model	CFI	TLI	RMSEA	χ^2^(*df*)
Single factor	0.34	0.19	0.19	4599.41 (170)^***^
2 factors (technology dependence + learning demands; work engagement + basic need satisfaction)	0.60	0.50	0.15	2884.58 (169)^***^
3 factors (technology dependence + learning demands; work engagement; basic need satisfaction)	0.66	0.58	0.13	2421.14 (167)^***^
4 factors (single factor for basic need satisfaction)	0.83	0.78	0.10	1299.98 (164)^***^
6 factors (separate factors for basic need satisfaction)	0.97	0.95	0.04	382.16 (155)^***^

df, degrees of freedom; ^***^p < 0.001.

*Technology dependence* was measured with a 4-item scale developed by Karr-Wisniewski and Lu (2010). All items were rated on a five-point Likert-type scale, ranging from 1 – totally disagree to 5 – totally agree. A sample item is: “When I do not have access to the information technology tools I use to support my job activities, this prevents me from being productive.”

*Learning demands* were measured with the intensified knowledge-related learning demands subscale from the Intensification of Job Demands Scale (Kubicek et al., 2015). The subscale consists of three items, rated on a five-point Likert-type scale, ranging from 1 – totally disagree to 5 – totally agree. A sample item is: “I have to acquire new expertise for the job more often.”

*Basic need* (i.e.*, autonomy, competence, relatedness*) *satisfaction* was measured with ten items obtained from Van den Broeck et al. (2010). All items were rated on a five-point Likert-type scale, ranging from 1—totally disagree to 5—totally agree. Three items were used to measure autonomy (sample item: “I feel like I can be myself at my job”), four items measured competence (sample item: “I feel competent at my job”), and three items were used to assess relatedness (sample item: “At work, I feel part of a group”).

*Work engagement* was measured with the Ultra-Short Utrecht Work Engagement Scale (UWES-3; Schaufeli et al., 2019). It consists of three items, rated on a seven-point Likert-type scale, ranging from 0—never to 7—always/every day. A sample item is: “At my work, I feel bursting with energy.”

### 2.3 Data analysis

We first performed a measurement model analysis with AMOS to ensure that the variables were clearly defined. The full information maximum likelihood (FIML) estimator was used to handle missing values. We evaluated and compared the fit of several measurement models, including a single-factor model, several multifactorial models that were theoretically justified, and a six-factor model in which all measured constructs were modelled as separate latent factors. Results presented in Table 1 showed the 6-factor model to be superior to other models with good fit indices (χ2 = 382,2, df = 155, *p* < 0.001; RMSEA = 0.04; CFI = 0.97), thus confirming the structural validity of our instruments.

We used SPSS and the PROCESS macro developed by Hayes (2018) for subsequent data analysis. List wise deletion was used to account for occasional missing data in these analyses. Where applicable, a bootstrapping approach with 5,000 bootstrap samples was used to estimate 95% confidence intervals. To examine the significant interactions more in detail, we estimated the effect of X on Y at the conditional values corresponding to the 16th and 84th percentiles of the moderator’s distribution, as suggested by Hayes (2018).

To test our study hypotheses, we performed conditional process analysis with Process (model 7), where technology dependence was included as an independent variable, work engagement—as a dependent variable, autonomy satisfaction—as a mediator, and learning demands—as a moderator. Since the satisfaction of all three basic needs is closely related (i.e., an autonomy-supportive work environment often helps to satisfy the need for competence and relatedness; Ryan and Deci, 2017), competence and relatedness were included as control variables in all regression equations. We also included key demographic characteristics (i.e., age, gender, managerial status, telecommuting, and contract type) as covariates.

## 3 Results

Pearson correlation coefficients between the main study variables are presented in Table 2. As expected, the satisfaction of the three basic needs were positively related. The satisfaction of basic needs, especially autonomy, was strongly positively correlated with work engagement. Demographic factors such as gender, age, managerial status, type of contract, and type of work arrangement (telework, hybrid, or office-based) were also related to the main study variables, which warrants their inclusion in further analyses as covariates. Among these factors, it is worth highlighting telework, which, as expected, was associated with greater dependence on technology, greater learning demands, and greater autonomy. Similarly, working from the office was related to lower technology dependence. Moreover, holding a managerial position was related to somewhat better opportunities to satisfy the needs for autonomy and competence, but not relatedness.

**Table 2:** Descriptive statistics and correlations between the study variables.

	*M*	SD	1	2	3	4
Technology dependence	3.9	1.0	(0.89)			
Learning demands	3.7	0.8	0.14^***^	(0.87)		
Autonomy S	3.8	0.8	0.05	0.13^***^	(0.74)	
Competence S	3.9	0.7	0.00	−0.08^*^	0.38^***^	(0.83)
Relatedness S	3.6	0.8	0.01	0.04	0.36^***^	0.28^***^
Work engagement	4.2	1.3	0.03	0.18^**^	0.53^***^	0.35^***^
Age	30.7	9.0	−0.04	0.10^**^	0.04	0.10^**^
Gender	1.7	0.4	−0.09^*^	0.02	−0.08*	−0.05
Manager	1.8	0.4	−0.08	−0.04	−0.13^***^	−0.18^***^
Contract	1.1	0.3	−0.04	−0.01	−0.05	−0.09*
Telework	0.6	0.5	0.26^***^	0.12^**^	0.14^***^	0.02
Office work	0.9	0.3	−0.16^***^	−0.06	0.03	−0.04

	5	6	7	8	9	10	11
Relatedness NS	(0.72)						
Work engagement	0.31^***^	(0.84)					
Age	−0.01	0.16^***^					
Gender	0.06	−0.01	−0.01				
Manager	−0.06	−0.17^***^	−0.17^***^	0.11^**^			
Contract	−0.07	−0.03	−0.04	−0.02	0.04		
Telework	0.02	−0.04	−0.01	−0.01	−0.03	−0.02	
Office work	0.08^*^	0.07	0.02	−0.02	−0.02	−0.04	−0.29^***^

Gender (1 = male, 2 = female), Manager (1 = manager, 2 = non-manager), Contract (1 = open-ended, 2 = fixed-term), telework (1 = only telework or hybrid, 0 = on-site work), office work (1 = on-site work or hybrid, 0 – only telework), S – satisfaction. Cronbach’s alpha coefficients are presented on the diagonal. ^*^p < 0.05; ^**^p < 0.01; ^***^p < 0.001.

**Figure 1:**
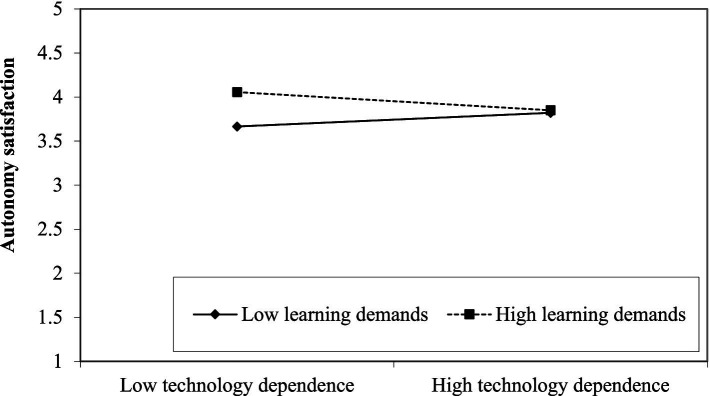
Simple slopes when predicting autonomy satisfaction.

We performed a conditional process analysis to test our hypotheses regarding the conditional effect of technology dependence on autonomy satisfaction and work engagement. Unstandardized and standardized regression coefficients are presented in Table 3. The results showed a significant interaction between technology dependence and learning demands when predicting autonomy satisfaction (*B* = −0.10; se = 0.03; *p* = 0.002), and the latter was also a significant predictor of work engagement. To examine this interaction effect more in detail, we tested the simple slopes among employees with low (16^th^ percentile) vs. high (84th percentile) learning demands (see Figure 1). In line with our expectations, the effect of technology dependence on autonomy satisfaction was positive (*B* = 0.07; se = 0.03; *p* = 0.03) when learning demands were low and negative (*B* = −0.09; se = 0.04; *p* = 0.02) when learning demands were high. Thus, our first hypothesis was supported. It is also worth noting that individuals with high learning demands but low dependence on technology were the most satisfied with their autonomy at work, as shown in Figure 1. Although the effect appears to be modest, this may be an interesting result that we will return to in the discussion section.

**Table 3:** Unstandardized and standardized regression coefficients.

Predictors	Dependent variable			
	Autonomy satisfaction		Work engagement	
	*B*(se)	β	*B*(se)	β
Technology dependence	0.36(0.11)^**^	0.50^**^	0.03(0.04)	0.03
Learning demands	0.50(0.12)^***^	0.56^***^		
Technology dependence × Learning demands	−0.10(0.03)^**^	−0.70^**^		
Age	0.00(0.00)	−0.03	0.01(0.00)^**^	0.10^**^
Gender	−0.11(0.06)	−0.07	0.06(0.09)	0.02
Manager	−0.02(0.06)	−0.01	−0.22(0.11)^*^	−0.07*
Contract	−0.07(0.10)	−0.02	−0.03(0.16)	−0.01
Telework	0.20(0.05)^***^	0.13^***^	0.11(0.09)	−0.04
Office work	0.23(0.09)^*^	0.09^*^	0.21(0.15)	0.05
Autonomy S			0.74(0.06)^***^	0.42^***^
Competence S	0.35(0.04)^***^	0.31^***^	0.29(0.07)^***^	0.15^***^
Relatedness S	0.21(0.03)^***^	0.23^***^	0.14(0.05)^*^	0.09^*^
*F* (*df*1; *df*2)	20.9 (11; 639)^***^		30.5 (10; 640)^***^	
*R* ^2^	0.26		0.32	
Δ*R*^2^	0.01		-	

Gender (1 = male, 2 = female), Manager (1 = manager, 2 = non-manager), Contract (1 = open-ended, 2 = fixed-term), telework (1 = only telework or hybrid, 0 = on-site work), office work (1 = on-site work or hybrid, 0 – only telework), S = satisfaction. B, unstandardized regression coefficient; β, standardized regression coefficient; ^*^p < 0.05; ^**^p < 0.01; ^***^p < 0.001.

To test our second hypothesis, we further inspected our model by comparing the conditional indirect effects of technology dependence on work engagement through autonomy satisfaction (see Table 4). Since we used the bootstrap approach suggested by Hayes (2018) to estimate the indirect effect, statistical significance was assessed based on 95% confidence intervals (an effect was considered statistically significant when its confidence interval did not include 0). As expected, this indirect effect was positive when learning demands were low (16th percentile) and negative when these demands were high (84th percentile). The significant conditional effect was also confirmed by the index of moderated mediation (index = −0.07; se = 0.02; 95% CI = [−0.12; −0.03]). Therefore, our second hypothesis was supported.

**Table 4:** Conditional indirect effects of technology dependence on work engagement.

Condition	Indirect effect	Boot se	Boot 95% CI
Low learning demands	0.05	0.02	[0.01; 0.10]
Average learning demands	−0.02	0.02	[−0.06; 0.02]
High learning demands	−0.07	0.03	[−0.13; −0.01]

Boot se, bootstrapped standard error; boot CI, bootstrapped confidence intervals.

## 4 Discussion

The advancement of digitalization is a fundamental factor shaping the modern world of work (Eurofound, 2021). It is already clear that the consequences of such changes will be diverse, as technologies make it possible to create workplaces that both satisfy and frustrate employees’ needs to a greater extent than ever before (Gagné et al., 2022). While new technologies can enable the achievement of previously unattainable results and support more flexible work arrangements, they can also displace employees from the labor market, limit their autonomy, and diminish their importance in organizations (World Economic Forum, 2023). Therefore, it is essential to understand what factors play a pivotal role in determining the impact of new technologies on employee well-being.

### 4.1 Implications for research

Our results revealed that dependence on technology at work (understood as the necessity of technology to perform work tasks) can be associated with both greater and lesser autonomy satisfaction, depending on subjectively perceived learning demands. Consistent with SDT (Ryan and Deci, 2017), employees who felt obliged to put in more effort to learn new things and were more dependent on technology to perform their work tasks experienced less autonomy satisfaction. Conversely, for employees who felt little pressure to update their skills and knowledge (i.e., those characterized by low learning demands), technology dependence served as an enabling factor associated with better autonomy.

Moreover, this tendency remained significant when predicting work engagement. In this case, technology dependence was positively related to work engagement when learning demands were low, but this relationship became negative when learning demands were high. These findings are not entirely unexpected. Work motivation is often analyzed using the conceptual framework of SDT, which unequivocally links the satisfaction of basic needs at work to the internalization of task motives (e.g., Autin et al., 2022). Similarly, various studies (e.g., Bakker and Oerlemans, 2019) reveal strong relationships between autonomy satisfaction and work engagement. Moreover, work engagement is a good predictor of productivity, as engaged employees put more energy into work and are more resilient in the face of drawbacks (Schaufeli and Bakker, 2010). Therefore, this result is significant because it highlights the critical role of learning demands in the performance of technology-dependent employees. In doing so, our study contributes to the theoretical understanding of the psychological mechanisms underlying technological advancements. One take-away from our findings is that the motivational dynamics of the interplay of dependence on technology and learning demands are of key importance, as they can strongly determine employees’ (sub)optimal functioning in the workplace.

The results of our study add to the growing body of literature that aims to explain the mixed effect of increasing dependence on technology. Parker and Grote (2022) insightfully observed that the impact of technology on employee autonomy could be varied and suggested employee skills and abilities among possible moderators of this effect. Similarly, drawing on a work design perspective, Wang et al. (2020) observed that work autonomy is strongly influenced by the use of information and communication technologies, and this effect is moderated by factors such as work-home segregation preferences, time management skills, employer control, and availability expectations. The results of our study supported the moderation assumption and provide new insights by identifying intensified learning demands as a potential factor shaping the effect of increasing technology dependence on employee well-being.

Furthermore, concerning learning demands, our study resonates with previous researchers’ insights into the emerging gap between employees who possess the necessary skills and those who have yet to acquire them (Parker and Grote, 2022). For employees equipped with the appropriate skills, emerging technologies can eliminate routine activities, provide opportunities to work at the time and place of choice, and improve working conditions in general. In contrast, employees whose skills do not match the evolving demands of the workplace may face negative consequences. These emerging technologies can exclude them from the labor market, reduce their workload, impose constant availability, or worsen their working conditions (Parent-Rocheleau and Parker, 2022; Sewell and Taskin, 2015; Spreitzer et al., 2017; Turja et al., 2024). Finally, as pointed out in the theoretical review by Gagné et al. (2022), the increasing use of technology at work can create additional job demands that, if not properly managed, may frustrate autonomy. This is consistent with our findings, which empirically demonstrated the moderating effect of one specific type of these additional demands.

It is worth noting that although in the current study the negative relationship between technology dependence and autonomy satisfaction only emerged when learning demands were high, the most autonomous respondents reported high learning demands but low technology dependence. Conversely, employees who were relatively technology-independent at work and had low learning demands reported the least autonomy satisfaction. Although our study design does not allow us to draw unequivocal conclusions, it appears that when the completion of work tasks is less dependent on technology, intensified learning demands function somewhat similarly to the concept of “new learning” (Decius et al., 2022). In other words, without the apparent necessity to apply technology in daily tasks, learning demands may not be perceived as a burden but rather as an opportunity to take advantage of technological solutions to perform work more efficiently. In future research, it would be valuable to examine this group of employees (i.e., those characterized by low technology dependence but high learning demands) in greater detail to better understand when technological mastery is perceived as a demand if the performance of work tasks does not depend on it. Based on SDT (Ryan and Deci, 2017; Ryan and Lynch, 1989), one might speculate that in such cases, the demands arise for reasons that are more aligned with the person’s long-term goals and values and, therefore, do not frustrate the person’s autonomy. Yet, other theoretical explanations are also possible, and this calls for more research on the topic.

### 4.2 Implications for practice

The findings of our study not only hold theoretical significance but also offer valuable insights for practical application. First, like other similar research (Bloom et al., 2014; Gagné et al., 2022; Lehdonvirta, 2018; Marsh et al., 2022; Wang et al., 2020), our results indicate a mixed impact of workplace technologies on employees’ basic needs and motivation. This ambivalence is an important message for managers overseeing technology integration into work processes. Although technological progress is often portrayed in an exclusively positive light, managers should be aware that technology-related changes can both enable their employees to perform their tasks more efficiently and frustrate their autonomy. Second, our study highlights the conditions under which workplace technologies hinder autonomy satisfaction. Specifically, managers should pay special attention to employees who still need to improve their skills to adapt to the evolving work environment despite their heavy reliance on technology. For this group, measures or interventions aimed at alleviating the frustration of autonomy (such as increased involvement in decision-making and opportunities to select preferred technologies) may be particularly beneficial. Finally, our research emphasizes that as technological solutions become increasingly embedded in work processes, the importance of training grows accordingly. Smart technologies are only effective when utilized by skilled employees who can fully harness the benefits of technological advancements. Therefore, investments in smart technology are unlikely to yield substantial returns unless organizations simultaneously invest in developing “smart” employees equipped with the necessary training and skills.

### 4.3 Limitations and future research guidelines

Several study limitations need to be taken into consideration when interpreting our results. First, the current study was based on a non-probability sample primarily composed of younger (average age around 31 years) white-collar employees. Although the analyzed statistical models were adjusted for the effects of background characteristics, the unequal representation of demographic groups limits the generalizability of our findings, particularly to older and lower-skilled workers. Therefore, additional research would be needed to determine whether these findings also apply to more diverse populations. The age factor may be particularly important, as ageism associated with technology use disproportionately affects older workers (Mariano et al., 2021). Moreover, given the negative relationship between workerskill level and the opportunities provided by technology (Dellot and Wallace-Stephens, 2017), blue-collar employees deserve separate attention from researchers. A second limitation is that the data were collected using self-report measures, meaning variables were subjectively evaluated. Although measurement model analyses confirmed that the assessed constructs are distinct from each other, we cannot rule out that responses may not entirely correspond to the objective work environment. This concern is especially relevant regarding technology dependence and learning demands, as their subjective perception may depend on factors not directly related to the objective need to assimilate and use technology (e.g., self-efficacy). Third, the study was based on cross-sectional data. While the direction of the investigated effects relies on strong theoretical assumptions, no causal inferences can be made based on our empirical findings. The relationship between learning demands and autonomy should be interpreted with particular caution. Although it is more in line with SDT to suggest that external demands frustrate autonomy, it is also possible that individuals with a greater autonomy satisfaction are more likely to internalize learning demands. Therefore, the relationship between these constructs may be reciprocal. As a result, it would be desirable to replicate these findings in a longitudinal study, which would allow testing not only the direction of the effects but also provide a more nuanced understanding of the dynamics of autonomy satisfaction in the context of technology. One particularly pertinent question to explore in future research is whether the frustration of autonomy is a stable phenomenon primarily caused by the chronic demand to master new things at work, or if it fluctuates over time. Finally, we must admit that intensified learning demands is a broad and, essentially, under researched phenomenon. It is likely an umbrella term encompassing various underlying reasons for the need to update one’s skills. Some of these reasons may be context-specific, influenced by an organization’s culture, its sector, or the country in which it operates. In sectors closely related to technological progress, such as IT and Fintech, the demands for intensive learning may be taken for granted and might not significantly shape employees’ reactions to advancing technologies. Additionally, it is important to note that this study was conducted in Lithuania, a country recognized for its rapid digitalization, increasing digital literacy, and security-oriented values (European Commission, 2024; World Values Survey, 2023). Therefore, future research should explore not only the heightened learning demands but also how these demands manifest and their impact across different cultural contexts.

## 5 Conclusion

Innovation in technology holds different implications for employees who have the necessary skills and those who feel the pressure to catch up. Our research revealed that for the former, it may be an empowering and motivating factor at work. However. for the latter, it poses a greater risk of becoming a servant of the technologies they use. As Parker and Grote (2022) suggest, there is a need to shift from a passive perspective focused on the adaptation of employees to technology, to a perspective that emphasizes the adaptation of technologies to align with employees’ competencies, needs, and goals. Efforts to coordinate employee training and technology implementation can largely determine who will serve whom, that is, whether technology will serve the people doing the work or vice versa.

## Data Availability

The raw data supporting the conclusions of this article will be made available by the authors, without undue reservation.
